# Pushing the age limit: Mitral valve surgery is safe and effective in octogenarians

**DOI:** 10.1016/j.jtcvs.2026.06.020

**Published:** 2026-07-06

**Authors:** Ricky Patil, Matthew Kazaleh, China Green, Jessica Woodford, Jeremy Wolverton, Matthew Romano, Steven F. Bolling, Robert B. Hawkins, Gorav Ailawadi

**Affiliations:** Department of Cardiac Surgery, University of Michigan, Ann Arbor, Mich.

**Keywords:** mitral regurgitation, mitral valve repair, mitral valve replacement, octogenarian

## Abstract

**Objective:**

Octogenarians represent a growing population of patients who require intervention for mitral valve disease. With the advent of transcatheter mitral approaches, the role of surgery, particularly valve repair, is under scrutiny. Comorbidity burden, poor tissue quality, and limited life expectancy have been cited as obstacles to benefiting from valve repair attempts. This study aims to capture outcomes of mitral valve surgery in octogenarians and compare outcomes of surgical valve repair and replacement.

**Methods:**

All patients aged 80 years and older who underwent surgical mitral valve operations at a high-volume valve center were included in analysis. Analysis of all patients and subanalysis of patients with degenerative mitral regurgitation was conducted. Outcomes evaluated included operative mortality and midterm survival.

**Results:**

Between 2015 and 2024, a total of 221 octogenarians underwent mitral surgery, including 158 (71%) undergoing mitral valve repair and 63 (29%) who underwent replacement for any etiology. Overall operative mortality was 5% (12 out of 221) (Society of Thoracic Surgeons Predicted Risk of Mortality, 5%) and was lower following mitral repair versus replacement (3% vs 11%; *P* = .04). On a subanalysis of 168 patients with degenerative mitral regurgitation (141 [84%] repair, 27 [16%] replacement), operative mortality was 1.2% (2 out of 168), with no difference by procedure type. Valve repair was associated with improved midterm survival for up to 4 years postsurgery (*P* = .02).

**Conclusions:**

Mitral surgery can be performed safely and effectively in appropriately selected octogenarians. Valve repair, when feasible, can promote improved midterm survival.

## CENTRAL MESSAGE

Surgical repair remains an effective first line therapy for octogenarian patients with DMR.

## Graphical Abstract

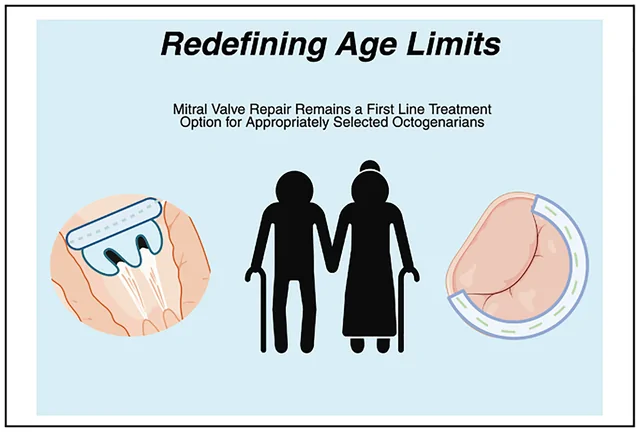

The role of mitral valve repair in octogenarians with degenerative MR remains under scrutiny.

As the average patient lifespan increases, the care of elderly patients becomes more pertinent to clinical practice. Per the US Census Bureau, the population of people older than age 80 years is expected to triple from 11.4 million in 2010 to 32.4 million in 2050.^1,2^ Furthermore, the prevalence of cardiovascular disease among octogenarians is as high as 85%, representing a major health care burden.^3^ The management of valvular disease in this population, particularly that of the mitral valve, remains uncertain. A 2006 Mayo Clinic study identified a 13.3% incidence of moderate or greater valvular disease in patients older than age 75 years.^4^ However, patients of advanced age are commonly perceived as too high risk to undergo conventional surgical treatment, with critics citing patient frailty, comorbidity burden, and disease complexity as obstacles to safe surgical intervention. Moreover, some heart teams have turned to newly available transcatheter mitral options as a first line approach before considering surgery in octogenarians.

Additionally, the utility of mitral valve repair in octogenarians remains debated.^5^ Valve repair for degenerative mitral disease has been demonstrated to improve long-term outcomes.^6–8^ However, it remains uncertain whether this survival benefit is realized in patients of advanced age. Furthermore, poor tissue quality and prolonged ischemia times have been cited as obstacles to safe execution of valve repair in elderly persons. As a result, valve repair is not pursued as aggressively in this patient subset.

The purpose of this study was to characterize short-term morbidity and mortality as well as long-term survival of mitral valve surgery in octogenarians at a high-volume valve center. Furthermore, we sought to compare outcomes of mitral valve repair versus replacement, for all patients and for patients presenting with primary degenerative mitral regurgitation (DMR).

## METHODS

The institutional review board of the University of Michigan approved the study protocol and publication of data (HUM00148119; April 2024). Patient data were extracted from the University of Michigan institutional component of the Society of Thoracic Surgeons (STS) Adult Cardiac Surgery Database and custom data extraction fields.

### Patients

All patients aged 80 years and older who underwent mitral surgery were included in analysis. Patients who received concomitant coronary artery bypass graft (CABG), tricuspid valve repair, and/or atrial fibrillation ablation procedure were also included. However, patients who received concomitant aortic valve/root/arch procedures, tricuspid valve replacement, transcatheter procedures, heart transplant, and/or ventricular assist devices were excluded from analysis. This cohort included patients with primary degenerative disease, functional disease, rheumatic disease, and endocarditis. Disease etiology was reported as documented in operative reports. No patients with previous mitral valve replacement were included in analysis. Subanalysis was pursued on all patients with primary DMR. This cohort was defined as all patients within the degenerative cohort with moderate or greater MR in the absence of severe mitral stenosis (MS). Primary outcomes studied were operative mortality and long-term survivorship. Secondary outcomes included STS-defined major morbidity, postoperative length of stay, intensive care unit length of stay, and discharge destination (Figure 1).

### Statistical Analysis

Categorical variables are represented as count (percent) and compared using χ^2^ analysis with 2-tailed Fisher exact text. Continuous variables are presented as a median (Q1, Q3) and compared by Wilcoxon rank-sum test. Risk scores, including the STS Predicted Risk of Mortality (STS-PROM) score and European System for Cardiac Operative Risk Evaluation II score, are presented as a mean ± SD and compared using Student *t* test.

Survival was analyzed using Kaplan-Meier analysis. Differences in survival curves were compared using the Log-rank test. Survival was reported at 1-, 2-, 3-, and 4-year follow-up. Recipients of valve repair and replacement varied drastically in disease etiology and valve anatomy, which could not be adjusted for due to elevated cohort heterogeneity. Furthermore, all cases were reviewed by high-volume mitral surgeons for repairability, introducing strong selection bias. Lastly, limited sample size prevented robust multivariable risk adjustment. Hence, adjusted analysis, although attempted, was not reported, with the acknowledgement that valve repair and replacement patients were inherently different groups. Rates of recurrent valve disease after surgery was also studied in the DMR cohort. All analyses were performed in Stata MP version 19 (StataCorp LLC)

## RESULTS

### Patient Characteristics and Operative Details

From January 2015 to September 2024, 221 patients aged 80 years or older received mitral surgery with or without concomitant tricuspid, maze, or CABG procedures. A total of 158 patients received repair (71%), whereas 63 patients underwent replacement (29%). The etiology of mitral disease included 187 (85%) patients with DMR, 20 (9%) patients with functional/secondary disease, 9 (4%) patients with rheumatic disease, and 5 (2%) patients with endocarditis (Table 1).

The median age of the entire cohort was 82 years (Q1, Q3, 81, 84 years) and did not differ by receipt of repair or replacement (Table 1). The mean STS-PROM for the group 5.0% ± 4.2%, with patients undergoing repair demonstrating significantly lower risk (*P* < .001). Valve repairs were done more often on female patients (*P* = .01). Additionally, those who underwent valve repair more often presented with moderate or greater MR, whereas recipients of valve replacement more often presented with moderate or greater MS. When looking at mitral disease etiology, those with degenerative disease more often underwent valve repair (*P* < .001), whereas patients presenting with rheumatic disease (*P* < .001) or endocarditis (*P* = .002) more often underwent replacement. Lastly, recipients of valve replacement more often had prior mitral valve repair (*P* < .001) and more often had a history of previous cardiac surgery (*P* < .001) (Table 1).

Intraoperatively, valve repair was able to be completed with both shorter crossclamp (*P* < .001) and bypass times (*P* = .01) compared with valve replacement, despite having higher rates of CABG (*P* = .33) and/or ablation procedures (*P* < .001† [*P* value was derived from all patients with preop atrial fibrillation]) (Table 2). Only 1 patient in the entire cohort had greater than mild mitral insufficiency during the immediate postoperative period.

### Early and Late Outcomes

Operative mortality was 5% (12 out of 221) (STS-PROM, 5.0% ± 4.2%) for the entire cohort. Recipients of valve repair demonstrated lower operative mortality compared with patients undergoing replacement (repair, 5 out of 158 [3%] vs replacement, 7 out of 63 [11%]; *P* = .04). For patients undergoing first-time mitral surgery without concomitant CABG, tricuspid surgery, or arrhythmia ablation, the operative mortality was 4% overall (2 out of 48), with both mortalities occurring after valve replacement.

In analysis of the entire cohort, recipients of repair also experienced less composite major morbidity (repair, 39 out of 158 [18%] vs replacement, 22 out of 63 [35%]; *P* < .001), including lower rates of postoperative stroke (*P* = .04), renal failure (*P* = .002), and prolonged ventilation (*P* = .01). Lastly, patients receiving valve repair spent less time in the intensive care unit (*P* < .001), and hospital following surgery (*P* < .001). Repair patients were more often discharged home instead of an extended care facility (*P* = .001) (Table 3).

After surgery, the median time of follow-up was 2.6 years. For the entire cohort, survival at 1, 2, 3, and 4 years was 87.8%, 84.7%, 77.4%, and 68.5%, respectively. For patients receiving valve repair, survival rates were 91.4%, 89.6%, 82.5%, and 72.4% at the respective time points compared with 79.1%, 72.8%, 65.3%, and 59.2%, respectively, for mitral replacement patients. Valve repair demonstrated a survival benefit compared with replacement (log rank *P* = .03) (Figure 2).

### Subanalysis of Patients With DMR

A total of 187 patients with primarily degenerative mitral disease were identified. In this cohort, 19 patients with significant mitral stenosis due to mitral annular calcification were identified and excluded from analysis. Thus, the final subset of patients was composed of 168 octogenarians with DMR, including 141 (84%) who received valve repair and 27 who underwent replacement (16%). The median patient age was 82 years (Q1, Q3, 81, 84 years). The mean STS-PROM was 4.3% ± 3.8%, with recipients of valve repair demonstrating lower risk compared with replacements (*P* < .001). Fifty-four patients (32%) presented with stage 3 or higher New York Heart Association functional class, with valve replacement recipients presenting with worse New York Heart Association functional class (*P* = .02). Additionally, recipients of replacement had higher rates of prior mitral surgery (*P* < .001). The most common valve primary pathology was posterior leaflet prolapse (n = 110 [65%]), followed by anterior leaflet (n = 23 [14%]) and bileaflet prolapse (n = 22 [13%]). Thirteen patients (8%) presented with degenerative disease with other form primary lesions, including either pure annular dilation or calcification. Recipients of valve repair more often presented with isolated posterior leaflet pathology (*P* < .001). Like the overall cohort, valve replacement was done more often in patients with previous cardiac surgery (*P* < .001) (Table E1). Valve repair in patients with DMR was achieved with significantly shorter crossclamp time (*P* = .02) and bypass time (*P* = .05) compared with replacements, despite having higher rates of CABG (*P* = .74) and maze procedures (*P* = .001) (Table E2).

Within the DMR cohort, operative mortality was low at 1.2% (2 out of 168) and was much lower than expected based on STS-PROM 4.3% ± 3.8%. There was no difference between repair and replacement. Recipients of repair demonstrated lower rates of major morbidity (*P* = .001), driven by higher rates of postoperative stroke (*P* = .006) and renal failure (*P* = .05) following replacement. Patients undergoing valve repair had shorter stays in the intensive care unit (*P* = .003), shorter postoperative lengths of stay (*P* = .005), and higher rates of being discharged home (*P* = .01) (Table E3).

Within the DMR cohort, survival at 1-, 2-, 3-, and 4-year follow-up was 96%, 94.3%, 87.6%, and 79.3%, respectively. For valve repair, survival was 97.6%, 96.6%, 89.6%, and 79.4%, respectively, whereas for valve replacement survival was 88.2%, 83.7%, 78.5%, and 78.5%, respectively. By log-rank test, valve repair demonstrated a significant survival benefit (*P* = .03). Survival curves appeared to converge after 4 years, but conclusions were limited by small sample size. Lastly, no difference in rates of recurrent mitral disease was observed on follow-up (*P* = .58) (Figure 3).

## DISCUSSION

In an era where transcatheter approaches have become the dominant approach in elderly patients, our experience based on 221 octogenarians showed that mitral surgery can be performed safely and effectively. Additionally, when anatomically feasible, valve repair can be performed with acceptable operative mortality, morbidity, and hospital resource utilization. Repair also conveys a survival benefit on midterm follow-up. In octogenarians with DMR, the outcomes were even better with an exceedingly low mortality (1.2%) compared with an STS-PROM of 4.3% and a low rate of postoperative complications. At 4-year follow-up, survival was almost 70% in all octogenarians and nearly 80% for the DMR cohort. Survival was greater for patients receiving valve repair.

Hesitancy to pursue surgery in the octogenarian population is partly due to the concern that limited life expectancy obviates any legitimate survival benefit. However, per reports from the Centers for Disease Control and Prevention, the life expectancy of an 80-year old is nearly 10 years.^9^ Moreover, the prevalence of mitral valve disease rises with age and is the greatest in the elderly, making appropriate management of this patient subset particularly relevant. A 2018 epidemiologic study from the Mayo Clinic that followed 1294 patients with moderate or greater MR reported a median age of diagnosis of 77 years.^10^ Those with moderate or greater primary regurgitation demonstrated a 63% 5-year and 41% 10-year mortality, which was significantly lower when compared with that of the general population.^10^ Only 15% of this population ultimately received surgery, with a bias toward younger patients. These findings highlight an important care deficit in the treatment of elderly patients. In appropriately selected patients, surgery can provide a safe treatment option for a potentially fatal condition.

The pursuit of valve repair over replacement can further improve survival outcomes in the octogenarian population. Although some earlier reported comparisons between valve repair and replacement in the elderly showed limited survival benefit, they demonstrated the importance of appropriate patient selection and prompt referral.^11–13^ The survival benefit associated with valve repair is limited in patients with non-DMR pathologies. Valve repair for endocarditis is beneficial over replacement provided adequate tissue is available after debridement. However, valves with extensive rheumatic disease or severe mitral annular calcification may not be amenable to durable repair. Ischemic MR may also lead to worse survival, irrespective of the type of surgery performed. In our 2008 study comparing mitral repair and replacement in patients older than age 75 years, ischemic MR was found to be independently associated with mortality (odds ratio, 3.9; *P* = .01).^11^ Hence, disease classification is important to determining the benefit of repair. Another factor limiting valve repairability is delay in referral. In fact, older guidelines recommended against surgery in elderly patients until the onset of symptoms.^14^ In contrast, an early surgery approach has demonstrated improved long-term survival and lower heart failure risk compared with initial medical management for patients with DMR.^15,16^ Hence, identifying what subset of patients benefit most from valve repair and pursuing prompt intervention is key to optimizing outcomes.

Our experience suggests that octogenarians with DMR represent a patient subset that also benefits from valve repair. Moreover, this was achieved with lower mortality, morbidity, and hospital resource utilization compared with replacements and at lower rates compared with what risk calculators would predict. Similar success has been observed in other reports. A 2011 study from the Mount Sinai group following 139 octogenarians with DMR identified valve replacement as an independent predictor of 90-day mortality (hazard ratio, 2.5; *P* = .045) and long-term mortality (*P* = .003).^17^ Likewise, the multicenter Mitral Valve Surgery in Octogenarians study, which followed 247 octogenarians with DMR also found valve repair to be associated with both lower 30-day mortality (*P* < .01) and better long-term survival (*P* = .0005).^18^ Additionally, in an analysis of 1040 octogenarian undergoing mitral surgery identified within the United Kingdom National Adult Cardiac Surgery Audit showed that recipients of mitral repair demonstrated lower in-hospital mortality compared with replacement (repair, 4.4% vs replacement, 8.3%; *P* < .001). This association retained significance on multivariable analysis (odds ratio, 2.01; *P* = .01).^19^ Hence, advanced age is not a barrier to valve repair.

Lastly, as percutaneous valve repair (transcatheter edge-to-edge repair [TEER]) becomes increasingly available, its role in relation to surgery requires further study, especially for elderly patients. Due to differences in risk profile and inherent selection bias, a comparison with transcatheter valve repair was not included in this analysis. Existing comparisons are limited in scope. Early randomized data suggest that surgery still provides greater freedom from recurrent valve regurgitation and/or reintervention on midterm follow-up.^20^ Retrospective comparisons have also shown better late survival with surgery in octogenarians.^21,22^ A 2025 Mayo Clinic study comparing TEER versus surgery in octogenarians demonstrated more recurrent MR after TEER compared with surgery (TEER, 33.3% vs surgery, 6.4%; *P* < .001; echocardiographic follow-up, 3.9 years) and reduced 5-year survival (TEER, 56% vs surgery, 68%; *P* = .019).^19^ For now, surgery remains the first line treatment for octogenarians, whereas transcatheter intervention is better suited for patients who are deemed too high risk.

### Limitations

This study has several limitations. First, the cohort examined is from a high-volume valve center, which limits generalizability to community practice. Additionally, because surgical candidacy is determined on an individual bases, the effect of selection bias must be acknowledged. Transcatheter therapy availability further contributes to this bias in this cohort because higher-risk patients may not be considered for surgery. Additionally, patients receiving valve repair and replacement present with inherently different risk profiles and levels of anatomic complexity. Hence, comparisons between these patient subsets should be interpreted in context.

## CONCLUSIONS

In modern clinical practice, mitral valve surgery for octogenarians can be performed safely and effectively for appropriately selected patients. Those with repairable disease anatomy showed better survival compared with those requiring valve replacement, especially in cases of pure degenerative regurgitation. Valve repair was more often pursued in a healthier cohort with amenable anatomy, whereas valve replacement cases were characterized by increased disease severity that precluded reconstruction. Overall, these findings show the benefit of surgical mitral repair in octogenarians in appropriately selected patients.

## Supplementary Material

1

## Figures and Tables

**FIGURE 1.**
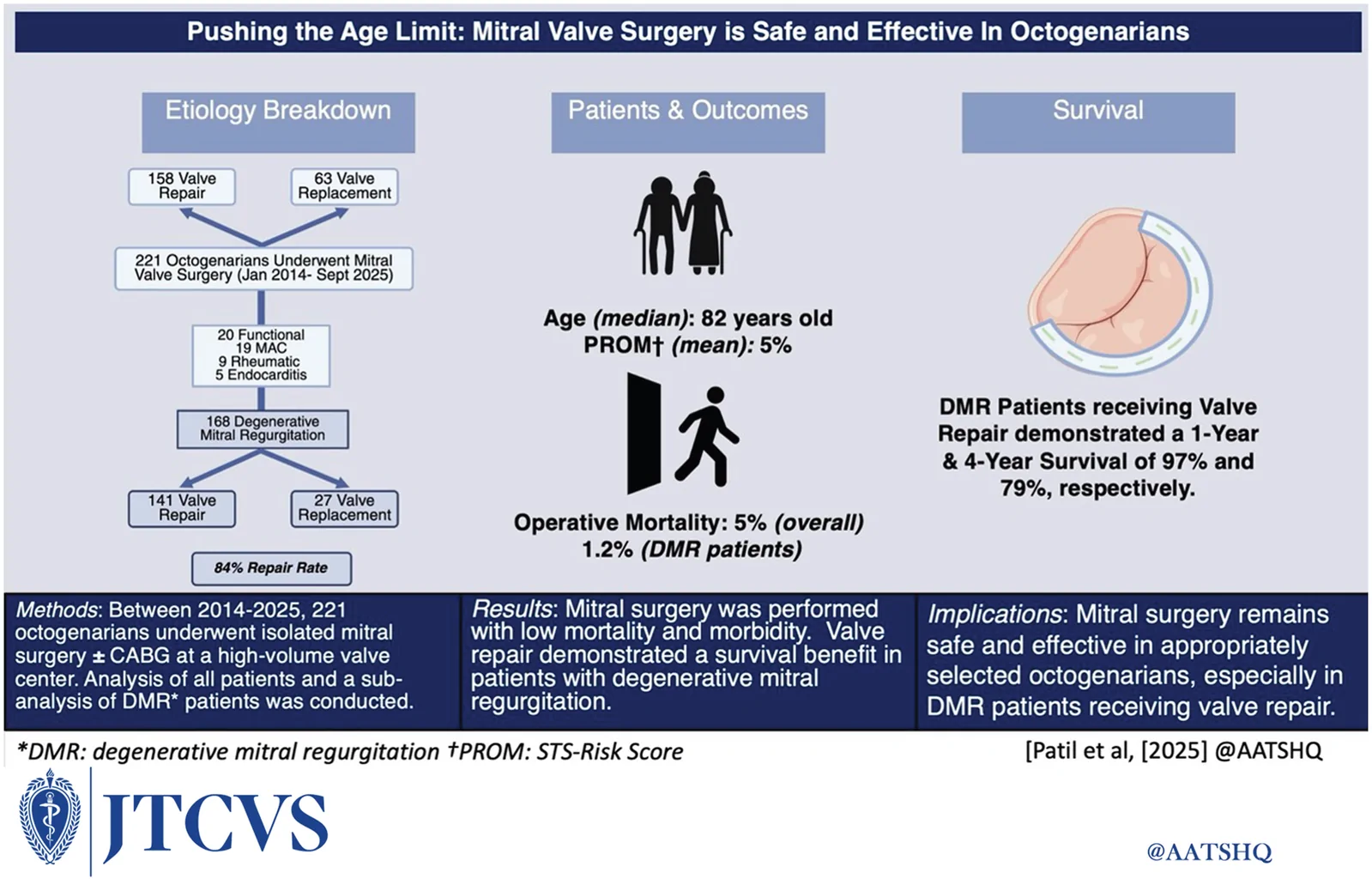
Graphical abstract showing etiology breakdown, outcomes, and long-term survival estimates. *MAC*, Mitral annular calcification; *DMR, degenerative mitral regurgitation; †*PROM*, Society of Thoracic Surgeons predicted risk of mortality.

**FIGURE 2.**
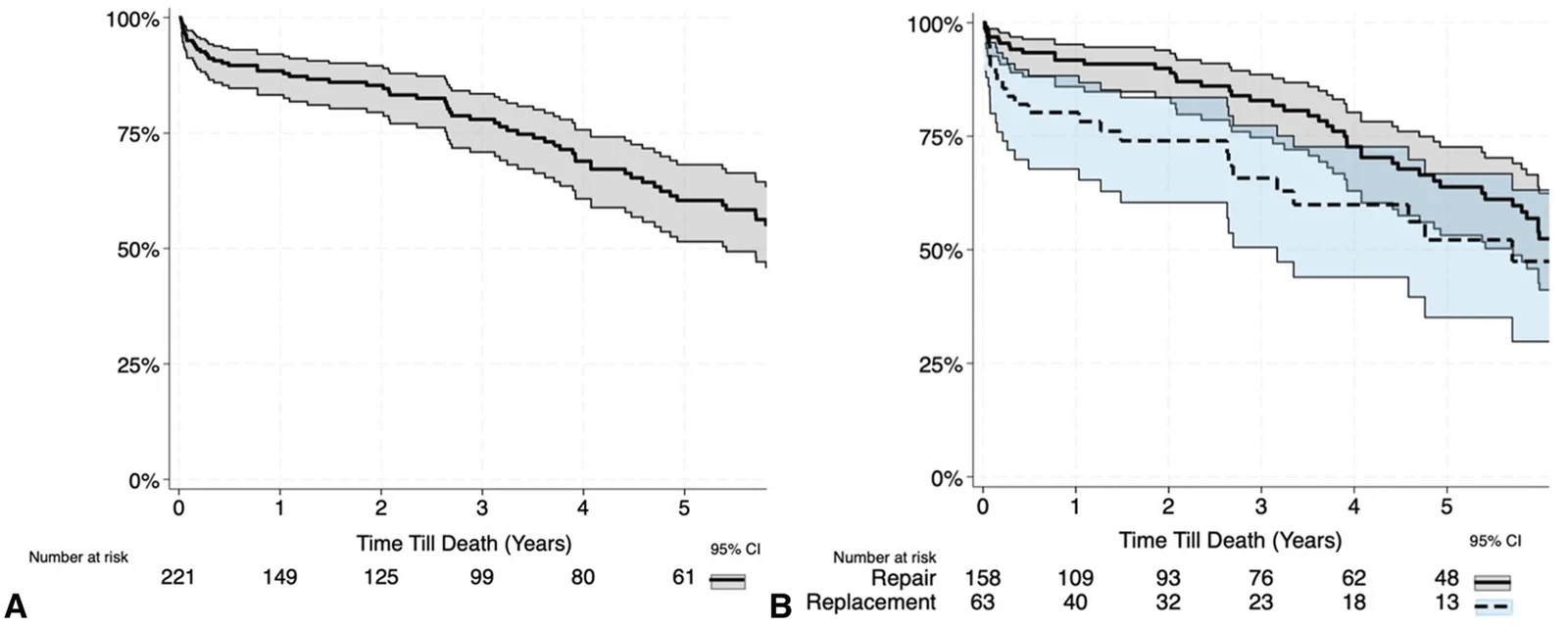
A, Overall, survival was 87.4%, 84.7%, 77.4%, and 68.5% at 1-, 2-, 3-, and 4-year follow-up, respectively. B, Recipients of valve repair demonstrated better survival compared with replacements (*P* = .02).

**FIGURE 3.**
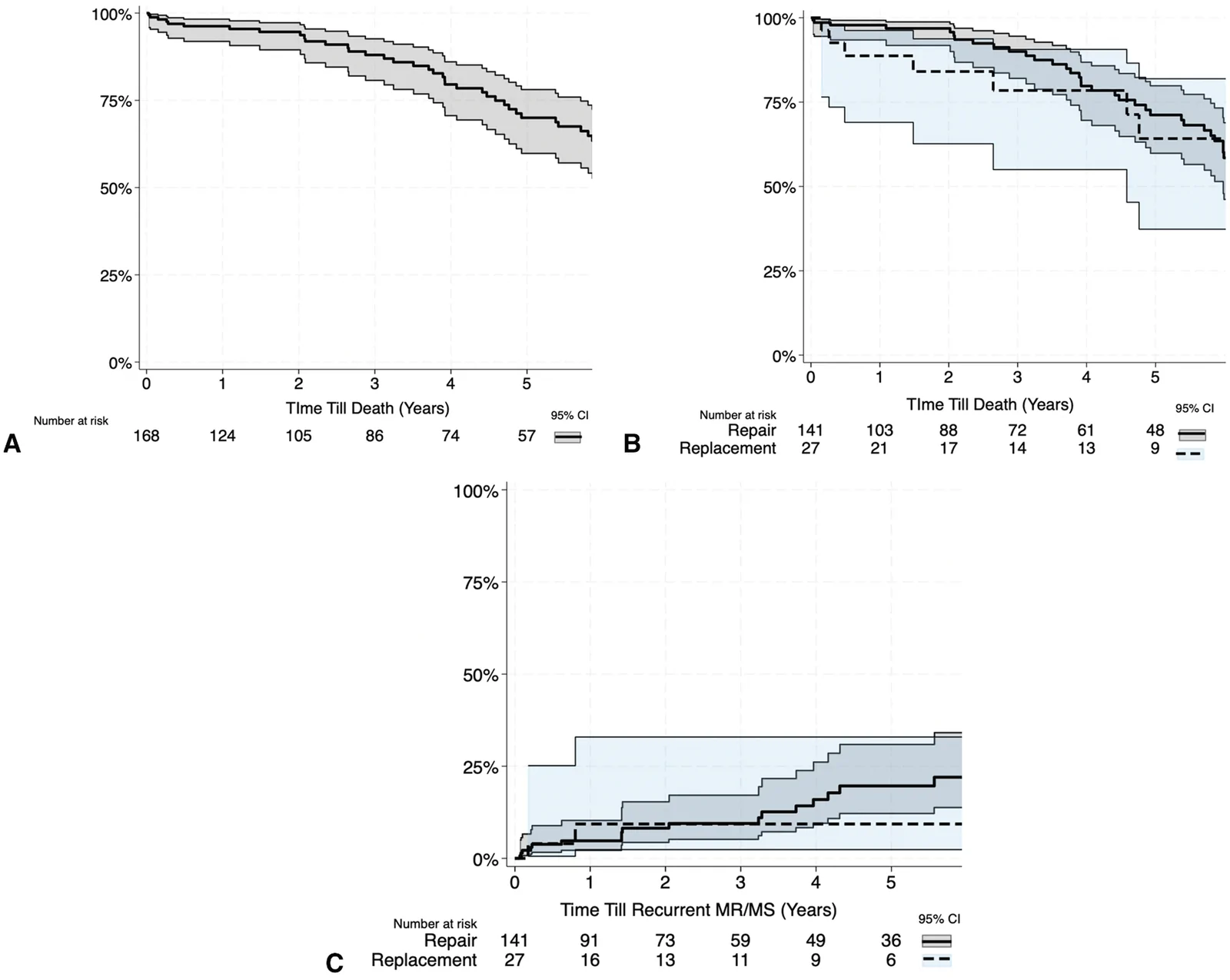
A, Within the degenerative mitral regurgitation cohort, survival was 96.0%, 94.3%, 87.6%, and 79.3% at 1-, 2-, 3-, and 4-year follow-up, respectively. B, Valve repair demonstrated improved survival compared with replacements on 4-year follow-up (*P* = .03). C, Rates of recurrent moderate or greater mitral regurgitation or stenosis did not reach significant difference (*P* = .58).

**TABLE 1.** Preoperative risk factors and procedural history for all patients

Preoperative characteristics	Overall (N = 221)	Repair (n = 158)	Replacement (n = 63)	Significance
Age (y)	82 (81–84)	82 (81–84)	80 (82–84)	.41
Female sex	112 (51)	89 (56)	23 (37)	.01*
Diabetes	46 (21)	29 (18)	17 (27)	.20
Dyslipidemia	127 (57)	90 (57)	37 (59)	.88
Hypertension	187 (85)	134 (85)	53 (84)	1.00
Atrial fibrillation	153 (69)	111 (70)	42 (67)	.63
Dialysis	0 (0)	0 (0)	0 (0)	1.00
Chronic lung disease	38 (17)	26 (16)	12 (19)	.69
Liver disease	7 (3)	6 (4)	1 (2)	.68
Stroke	20 (9)	10 (6)	10 (16)	.04
Prior myocardial infarction	58 (26)	41 (26)	17 (27)	.87
NYHA functional class III or IV	76 (34)	46 (29)	30 (48)	.01*
STS-PROM (%)	5.0 ± 4.2	4.0 ± 3.6	7.4 ± 4.7	<.001*
EuroSCORE II score (%)	12.1 ± 0.1	11.9 ± 0.1	14.9 ± 0.1	.003*
Baseline lab markers				
Creatinine (mg/dL)	1.04 (0.88–1.22)	1.05 (0.87–1.25)	1.04 (0.89–1.2)	.19
Bilirubin (mg/dL)	0.8 (0.5–1)	0.8 (0.6–1)	0.7 (0.5–1)	.13
INR	1.1 (1–1.1)	1.1 (1–1.1)	1.1 (1–1.3)	.14
Hemodynamics				
Ejection fraction (%)	60 (55–65)	60 (55–65)	60 (55–65)	.27
≥+2 Moderate MR	201 (91)	158 (100)	43 (68)	<.001*
≥+2 Moderate MS	28 (13)	2 (1)	26 (41)	<.001*
Mitral disease etiology				
Degenerative	187 (85)	142 (90)	45 (71)	<.001*
Functional	20 (9)	15 (9)	5 (8)	.80
Rheumatic	9 (4)	1 (1)	8 (13)	<.001*
Endocarditis	5 (2)	0 (0)	5 (8)	.002*
Procedural history				
Previous PCI	47 (21)	30 (19)	17 (27)	.21
Incidence				
First surgery	179 (81)	140 (89)	39 (62)	<.001*
First reoperation	36 (16)	14 (9)	22 (35)	
Second/third reoperation	6 (3)	4 (2)	2 (3)	
Previous CABG	23 (10)	13 (8)	10 (16)	.14
Previous mitral valve repair procedure	24 (11)	4 (3)	24 (38)	<.001*

Values are presented as n (%), mean ± SD, or median (interquartile range). *NYHA*, New York Heart Association; *STS-PROM*, Society of Thoracic Surgeons predicted Risk of Mortality; *EuroSCORE*, European System For Cardiac Operative Risk Evaluation; *INR*, international normalized ratio; *MR*, mitral regurgitation; *MS*, mitral stenosis; *PCI*, percutaneous coronary intervention; *CABG*, coronary artery bypass graft.

* *P* < .05.

**TABLE 2.** Procedural attributes for the entire cohort

Intraoperative characteristic	Overall	Repair	Replacement	Significance
Adjunct procedures				
Tricuspid repair*	83 (73)	67 (77)	(59)	.09
Coronary artery bypass graft	23 (10)	19 (12)	4 (6)	.33
Atrial fibrillation ablation†	114 (75)	93 (84)	21 (50)	<.001‡
Cardiopulmonary bypass time	97 (77–131)	92.5 (75–124)	107 (85–149)	.01‡
Crossclamp time§	75 (58–103)	71 (57–96)	91.5 (71–128)	<.001‡
Nonelective status	15 (7)	5 (3)	10 (16)	.002‡
Operative approach				.009‡
Sternotomy	185 (84)	139 (88)	46 (73)	
Thoracotomy	36 (16)	19 (12)	17 (27)	

Values are presented as n (%) or median (interquartile range).

* One hundred fourteen out of 221 patients who had moderate or greater tricuspid regurgitation were included in analysis.

† Ablation rates were calculated out of all patients with preoperative atrial fibrillation (n = 153).

‡ *P* < .05.

§ Twenty-six patients who underwent beating heart surgery were excluded.

**TABLE 3.** Postoperative outcomes for all patients

Postoperative characteristic	Overall	Repair	Replacement	Significance
Operative mortality	12 (5)	5 (3)	7 (11)	.04*
Composite morbidity	39 (18)	17 (11)	22 (35)	<.001*
Stroke	8 (4)	3 (2)	5 (8)	.04*
Renal failure	15 (7)	5 (3)	10 (16)	.002*
Dialysis	9 (4)	3 (2)	6 (10)	.02*
Prolonged ventilation	26 (12)	13 (8)	13 (21)	.01*
Reoperation	6 (3)	3 (2)	3 (5)	.36
Atrial fibrillation	63 (29)	43 (27)	20 (32)	.51
Cardiac arrest	11 (5)	4 (3)	7 (11)	.01*
Intensive care unit length of stay (h)	73.6 (46–125.4)	58.6 (32.7–109.2)	95 (71.1–178.3)	<.001*
Postoperative length of stay (d)	7 (5–11)	7 (5–10)	10 (7–16)	<.001*
Discharged home	141 (64)	112 (71)	29 (46)	.001*

Values are presented as n (%) or median (interquartile range).

* *P* < .05.

## PERSPECTIVE

The role of surgery in the treatment of mitral disease in octogenarians is under scrutiny, due to preconceptions of elevated risk. In fact, some heart teams turn to transcatheter treatment as a first-line therapy. However, our experience demonstrates that surgery can be performed safely in appropriately selected octogenarians and that surgical valve repair can still provide a survival benefit.

## Abbreviations and Acronyms

CABG: coronary artery bypass graft
DMR: degenerative mitral regurgitation
MS: mitral stenosis
STS-PROM: Society of Thoracic Surgeons Predicted Risk of Mortality
TEER: transcatheter edge-to-edge repair

## References

[1] Kodali SK, Velagapudi P, Hahn RT, Abbott D, Leon MB. Valvular heart disease in patients ≥80 years of age. J Am Coll Cardiol. 2018;71(18):2058–2072. 10.1016/j.jacc.2018.03.459 29724358

[2] Vincent GK, Victoria AV. THE NEXT FOUR DECADES, The Older Population in the United States: 2010 to 2050, Current Population Reports, 25–1138. Washington, DC: U.S. Census Bureau; 2010.

[3] Qu C, Liao S, Zhang J, Burden of cardiovascular disease among elderly: based on the Global Burden of Disease Study 2019. Eur Heart J Qual Care Clin Outcomes. 2024;10(2):143–153. 10.1093/ehjqcco/qcad033 PMC10904724 37296238

[4] Nkomo VT, Gardin JM, Skelton TN, Gottdiener JS, Scott CG, Enriquez-Sarano M. Burden of valvular heart diseases: a population-based study. Lancet. 2006;368(9540):1005–1011. 10.1016/S0140-6736(06)69208-8 16980116

[5] Thourani VH, Weintraub WS, Guyton RA, Outcomes and long-term survival for patients undergoing mitral valve repair versus replacement: effect of age and concomitant coronary artery bypass grafting. Circulation. 2003; 108(3):298–304. 10.1161/01.CIR.0000079169.15862.13 12835220

[6] David TE, Ivanov J, Armstrong S, Christie D, Rakowski H. A comparison of outcomes of mitral valve repair for degenerative disease with posterior, anterior, and bileaflet prolapse. J Thorac Cardiovasc Surg. 2005;130(5):1242–1249. 10.1016/j.jtcvs.2005.06.046 16256774

[7] Gillinov AM, Cosgrove DM, Blackstone EH, Durability of mitral valve repair for degenerative disease. J Thorac Cardiovasc Surg. 1998;116(5): 734–743. 10.1016/S0022-5223(98)00450-4 9806380

[8] Suri RM, Schaff HV, Dearani JA, Survival advantage and improved durability of mitral repair for leaflet prolapse subsets in the current era. Ann Thorac Surg. 2006;82(3):819–826. 10.1016/j.athoracsur.2006.03.091 16928491

[9] Arias E, Xu JQ, Kochanek K. United States life tables, 2023. Natl Vital Stat Rep. 2025;74(6):1–63. 10.15620/cdc/174591 PMC12926848 41661677

[10] Dziadzko V, Clavel MA, Dziadzko M, Outcome and undertreatment of mitral regurgitation: a community cohort study. Lancet. 2018;391(10124): 960–969. 10.1016/S0140-6736(18)30473-2 PMC5907494 29536860

[11] Ailawadi G, Swenson BR, Girotti ME, Is mitral valve repair superior to replacement in elderly patients? Ann Thorac Surg. 2008;86(1):77–86. 10.1016/j.athoracsur.2008.03.020 18573402

[12] Nloga J, Hénaine R, Vergnat M, Mitral valve surgery in octogenarians: should we fight for repair? A survival and quality-of-life assessment. Eur J Cardiothorac Surg. 2011;39(6):875–880. 10.1016/j.ejcts.2010.11.019 21177117

[13] Pavlopoulos D, Mylonas KS, Avgerinos D, Mitral valve surgery in octogenarians: identifying factors that can guide clinical decision-making. Asian Cardiovasc Thorac Ann. 2023;31(6):498–505. 10.1177/02184923231187681 37431635

[14] American College of Cardiology/American Heart Association Task Force on Practice Guidelines, Society of Cardiovascular Anesthesiologists, Society for Cardiovascular Angiography and Interventions, Society of Thoracic Surgeons, Bonow RO, Carabello BA, Kanu C, ACC/AHA 2006 guidelines for the management of patients with valvular heart disease: a report of the American College of Cardiology/American Heart Association Task Force on Practice Guidelines (writing committee to revise the 1998 guidelines for the Management of Patients with Valvular Heart Disease): developed in collaboration with the Society of Cardiovascular Anesthesiologists: endorsed by the Society for Cardiovascular Angiography and Interventions and the Society of Thoracic Surgeons. Circulation. 2006;114(5):e84–e231. 10.1161/CIRCULATIONAHA.106.176857 16880336

[15] Suri RM, Vanoverschelde JL, Grigioni F, Association between early surgical intervention vs watchful waiting and outcomes for mitral regurgitation due to flail mitral valve leaflets. JAMA. 2013;310(6):609–616. 10.1001/jama.2013.8643 23942679

[16] Kang DH, Park SJ, Lee SA, Early surgery or conservative care for asymptomatic aortic stenosis. N Engl J Med. 2020;382(2):111–119. 10.1056/NEJMoa1912846 31733181

[17] Chikwe J, Goldstone AB, Passage J, A propensity score-adjusted retrospective comparison of early and mid-term results of mitral valve repair versus replacement in octogenarians. Eur Heart J. 2011;32(5):618–626. 10.1093/eurheartj/ehq331 20846993

[18] Chivasso P, Bruno VD, Farid S, Predictors of survival in octogenarians after mitral valve surgery for degenerative disease: the Mitral Surgery in Octogenarians study. J Thorac Cardiovasc Surg. 2018;155(4):1474–1482.e2. 10.1016/j.jtcvs.2017.11.027 PMC5864964 29249501

[19] Burns DJP, Angelini GD, Benedetto U, Caputo M, Ciulli F, Vohra HA. Early mortality and neurologic outcomes following mitral valve surgery in the very elderly. J Card Surg. 2022;37(12):4510–4516. 10.1111/jocs.17098 36335608

[20] Feldman T, Kar S, Elmariah S, Randomized comparison of percutaneous repair and surgery for mitral regurgitation: 5-year results of EVEREST II. J Am Coll Cardiol. 2015;66(25):2844–2854. 10.1016/j.jacc.2015.10.018 26718672

[21] Danesh S, Schaff HV, Holst KA, Transcatheter edge-to-edge repair versus mitral valve surgery in octogenarians: comparative analysis of safety, durability, and survival. J Thorac Cardiovasc Surg. 2026;171:110–120.e6. 10.1016/j.jtcvs.2025.08.016 40849062

[22] Chikwe J, Chen Q, Bowdish ME, Surgery and transcatheter intervention for degenerative mitral regurgitation in the United States. J Thorac Cardiovasc Surg. 2025;169(1):80–88.e19. 10.1016/j.jtcvs.2024.01.014 38237762

