# Supplementary material for: Pushing the age limit: Mitral valve surgery is safe and effective in octogenarians
Source: J Thorac Cardiovasc Surg. Author manuscript; Available in PMC 2026 Oct 6. (PMC13636366; doi:10.1016/j.jtcvs.2026.06.020)
Supplement: Supplementary file 1 [file NIHMS2208893-supplement-1.pdf]

TABLE E1. Preoperative comorbidity burden, select lab values, hemodynamics, and procedural history for degenerative mitral regurgitation patients

Preoperative characteristics	Overall (N = 168)	Repair (n = 141)	Replacement (n = 27)	Significance
Age (y)	82 (81-84)	82 (81-84)	82 (80-84)	.44
Female sex	75 (45)	61 (43)	14 (52)	.52
Diabetes	30 (18)	25 (18)	5 (19)	1.00
Dyslipidemia	93 (55)	77 (55)	16 (59)	.68
Hypertension	139 (83)	118 (84)	21(78)	.42
Atrial fibrillation	115 (68)	97 (69)	18 (67)	.83
Dialysis	0	0 (0)	0 (0)	1.00
Chronic lung disease	30 (18)	24 (17)	6 (22)	.58
Liver disease	7 (4)	6 (4)	1 (4)	1.00
Stroke	9 (5)	8 (6)	1 (4)	1.00
Prior myocardial nfarction	43 (26)	37 (26)	6 (22)	.81
NYHA functional class III or IV	54 (32)	40 (28)	14 (52)	.02*
STS-PROM (%)	4.3 ± 3.8	3.8 ± 3.6	6.8 ± 4.2%	<.001*
EuroSCORE II score (%)	11.1% ± 8.5	10.4% ± 8.4	14.2% ± 8.2	.03*
Baseline lab markers				
Creatinine (mg/dL)	1.05 (0.89, 1.22)	1.04 (0.87, 1.22)	1.09 (0.99, 1.2)	.20
Bilirubin (mg/dL)	0.8 (0.6, 1)	0.8 (0.6, 1)	1.0 (0.7, 1.1)	.13
INR	1.1 (1, 1.1)	1.1 (1, 1.1)	1.1 (1, 1.5)	.30
Hemodynamics				
Ejection fraction (%)	60 (55, 65)	60 (55, 65)	60 (55, 65)	.57
Valve pathology incidence				
Posterior leaflet prolapse	110 (65%)	100 (71%)	10 (37%)	<.001*
Anterior leaflet prolapse	23 (14%)	20 (14%)	3 (11%)	
Bileaflet prolapse	22 (13%)	17 (12%)	5 (19%)	
Other	13 (8%)	4 (3%)	9 (33%)	
Procedural history				
Incidence				<.001*
First surgery	143 (85%)	129 (91%)	14 (52%)	
First reoperation	22 (13%)	10 (7%)	12 (44%)	
Second reoperation	3 (2%)	2 (2%)	1 (4%)	
Previous PCI	32 (19%)	26 (18%)	6 (22%)	.60
Previous CABG	12 (7%)	7 (5%)	5 (19%)	.15
Previous mitral valve repair	15 (9%)	4 (3%)	11 (41%)	<.001*

Values are presented as n (%), mean ± SD, or median (interquartile range). *NYHA*, New York Heart Association; *STS-PROM*, Society of Thoracic Surgeons predicted Risk of Mortality; *EuroSCORE*, European System For Cardiac Operative Risk Evaluation; *INR*, international normalized ratio; *MR*, mitral regurgitation; *MS*, mitral stenosis; *PCI*, percutaneous coronary intervention; *CABG*, coronary artery bypass graft. *$P < .05$.

ADULT

TABLE E2. Procedural attributes for the DMR cohort, recipients of mitral repair, and recipients of mitral replacements

Intraoperative characteristics	Overall	Repair	Replacement	Significance
Adjunct procedures				
Tricuspid repair†	69 (70)	58 (78)	11 (85)	1.0
Coronary artery bypass graft	19 (11)	17 (12)	2 (7)	.74
Atrial fibrillation ablation‡	89 (77)	81 (84)	8 (44)	.001*
Cardiopulmonary bypass time	96 (76-129)	92 (75-120)	106 (89-149)	.05*
Crossclamp time§	73 (58-99)	71 (55-95)	92 (69-126)	.02*
Nonelective status	7 (4)	5 (3)	2 (7)	.29
Operative approach				.01*
Sternotomy	146 (87)	127 (90)	19 (70)	
Thoracotomy	22 (13)	14 (10)	8 (30)	

Values are presented as n (%) or median (interquartile range). *$P < .05$. †One hundred fourteen out of 221 patients who had moderate or greater tricuspid regurgitation were included in analysis. ‡Ablation rates were calculated out of all patients with preoperative atrial fibrillation (n = 153). §Twenty-six patients who underwent beating heart surgery were excluded.

ADULT

TABLE E3. Postoperative outcomes for all degenerative mitral regurgitation patients, recipients of mitral repair, and recipients of mitral replacement

Postoperative characteristics	Overall	Repair	Replacement	Significance
Operative mortality	2 (1)	2 (1)	0 (0)	1.0
Composite morbidity	19 (11)	10 (7)	9 (33)	.001*
Stroke	6 (4)	2 (1)	4 (15)	.006*
Renal failure	6 (4)	3 (2)	3 (11)	.05*
Dialysis	2 (1)	1 (1)	1 (4)	.30
Prolonged ventilation	11 (7)	7 (5)	4 (15)	.08
Reoperation	4 (2)	4 (3)	0 (0)	1.00
Atrial fibrillation	44 (26)	38 (27)	6 (22)	.81
Cardiac arrest	4 (2)	2 (1)	2 (7)	.12
Intensive care unit length of stay (h)	71 (38-114)	55 (30-105)	91 (70-152)	.003*
Postoperative length of stay (d)	7 (5-10)	6 (5-9)	8 (7-14)	.005*
Discharged home†	118 (71)	105 (74)	13 (48)	.01*

Values are presented as n (%) or median (interquartile range). *$P < .05$. †Excludes 2 patients who died in the hospital.
